# Rückenschmerz: ein Phänomen des Alters? Degenerative Veränderungen an der Wirbelsäule gelten mit zunehmendem Alter als normal. Aber wie ist dieses „Normal“ im Alter definiert, führt es zwangsweise zu mehr Beschwerden und einer relevanten Einschränkung der Lebensqualität?

**DOI:** 10.1007/s00391-021-01912-9

**Published:** 2021-06-11

**Authors:** Christoph Alexander Stueckle, Sarah Talarczyk, Kerstin Frauke Stueckle, Christian Beisenherz, Patrick Haage

**Affiliations:** 1 Faculty of Health, University Witten/Herdecke, Witten, Deutschland; 2 Diagnostic and Interventional Radiology, HELIOS University Hospital Wuppertal, University Witten/Herdecke, Wuppertal, Deutschland; 3 MR Imaging Institute, Dr. Amirfallah, Europaplatz 11, 44269 Dortmund, Deutschland; 4 Medical Center MVZ Professor Uhlenbrock & Partner GmbH, Dortmund, Deutschland; 5 Deutsche Rentenversicherung, Knappschaft Bahn, See, Bochum, Deutschland

**Keywords:** Spezifischer Rückenschmerz, Degeneration, Geriatrie, Outcome, Morphologische Veränderungen, Specific back pain, Degeneration, Geriatrics, Outcome, Morphological changes

## Abstract

**Hintergrund:**

Unter der Vorstellung, dass ein gewisses Maß an Degeneration im Alter normal ist, sollten die Veränderungen erkannt werden, die signifikant zu Beschwerden führen. Es sollte sichergestellt werden, dass der geriatrische Patient adäquat behandelt wird und schnell wieder in sein normales, schmerzfreies Leben zurückfinden kann.

**Methodik:**

Durchgeführt wurde eine prospektive Untersuchung an symptomatischen Patienten, die zu einer MR-Untersuchung der Wirbelsäule kamen. Es wurden das Vorliegen einer Spinalkanalstenose, osteochondrotische und spondylarthrotische Veränderungen sowie Nervenwurzelaffektionen beurteilt. In einem Kurzinterview wurden die Beeinträchtigungen im Alltag, Dauer der Beschwerden bis zur Kontaktaufnahme mit dem Arzt und daraus resultierende Beeinträchtigungen erhoben. Die Ergebnisse wurden mit dem Alter, der Gruppe der Patienten unter und über 65 Lebensjahre sowie mit dem Schmerzscore korreliert.

**Ergebnisse:**

Das Alter ist signifikant positiv korreliert mit der Facettengelenkarthrose, spinaler Einengung, osteochondrotischen Veränderungen und der intraforaminalen Nervenwurzelaffektion. Es zeigt sich keine signifikante Korrelation zwischen Schmerzscore und Lebensalter.

Der Schmerzscore zeigt eine signifikante Korrelation für die Nervenwurzelaffektion, Facettengelenkarthrose und spinale Enge. Die Schmerzdauer ist beim älteren Patienten signifikant kürzer, bis er den behandelnden Arzt aufsucht, während die Beeinträchtigungen im Alltag signifikant stärker ausgeprägt sind.

**Schlussfolgerung:**

Das Alter selbst ist nicht mit Schmerzempfinden korreliert. Isolierte Merkmale wie Nervenwurzelaffektion und Facettengelenkarthrose zeigen eine positive Korrelation zum Schmerz. Der ältere Patient geht schneller zum Arzt, da er den Rückenschmerz als Einschränkung seines täglichen Lebens empfindet.

## Abstract (en)

**Background:**

Under the assumption that a certain degree of degeneration is normal in old age, the changes that significantly lead to discomfort should be identified. Care should be taken to ensure that the geriatric patient receives adequate treatment and can quickly return to a normal pain-free life.

**Material and methods:**

A prospective study was performed on symptomatic outpatients who came for a magnetic resonance (MR) examination of the spine. The presence of spinal stenosis, osteochondrotic and spondylarthrotic changes and nerve root affections were assessed. A brief interview was conducted to assess impairment of daily life, duration of symptoms until contact with the physician, and resulting impairment. The results were correlated with age, the groups of patients under and over 65 years of age, and the pain score.

**Results:**

Age is significantly positively correlated with facet joint arthrosis, spinal stenosis, osteochondrotic changes and intraforaminal nerve root affection. There is no significant correlation between the pain score and age.

The pain score shows significant correlation to nerve root affection, facet joint osteoarthritis and spinal constriction. The duration of pain until a visit to the treating physician is significantly shorter in older patients, while the impairments in everyday life are significantly more pronounced under the existing back pain.

**Conclusion:**

Age itself is not correlated with pain perception. Isolated features such as nerve root affection and facet joint arthrosis show a positive correlation with the pain. The older patient goes to the doctor more quickly because the back pain is perceived as a restriction of daily life.

Der demografische Wandel unserer Gesellschaft führt zu einer durchschnittlich steigenden Lebenserwartung, gleichzeitig sind viele Menschen bis ins hohe Alter auch sportlich aktiv. Der Rückenschmerz, als eine der häufigsten orthopädischen Diagnosen überhaupt, betrifft Menschen aller Altersgruppen. Um eine zielgerichtete und wirksame Therapie anbieten zu können, ist es wichtig zu wissen, welche Veränderungen gerade beim älteren Menschen zu einer signifikanten Beschwerdesymptomatik führen und welche Veränderungen regelhaft mit dem Alter vorkommen, ohne Beschwerden zu verursachen. Wegen der Gefahr einer Chronifizierung ist es wichtig, den Rückenschmerz richtig einzuordnen und richtig zu behandeln. Rückenschmerz, der das Leben einschneidend verändert, kann und muss behandelt werden. Eine erfolgreiche Behandlung sollte den Patienten im besten Falle wieder zu einem zufriedenen, schmerzfreien Leben zurückführen.

Die berichtete Lebenszeitprävalenz des Rückenschmerzes schwankt zwischen 20 und 40 % [16]. Die Einschränkungen für die Patienten sind in der akuten Episode und bei Chronifizierung der Beschwerden deutlich ausgeprägt und zeigen eine deutliche interindividuelle Bandbreite. Der akute Rückenschmerz ist bezüglich seiner Prognose unsicher; in etwa der Hälfte aller Fälle klingt er innerhalb von 6 Wochen ab [4]. Andererseits zeigen Untersuchungen, dass 62 % der Betroffenen 12 Monate später immer noch Schmerzen haben [17].

In Deutschland wird zwischen spezifischem und nichtspezifischem Rückenschmerz unterschieden. Der Definition nach ist beim spezifischen Rückenschmerz ein pathoanatomischer Grund für die Beschwerdesymtomatik nachweisbar, also eine bildmorphologisch fassbare Veränderung, während beim nichtspezifischen Rückenschmerz kein morphologisches Korrelat für die Beschwerden zu finden ist [21].

Es ist allgemein akzeptiert, dass sich das Skelettsystem und auch die Wirbelsäule im Laufe des Lebens verändern. Allerdings gibt es nur relativ wenige Untersuchungen, die überhaupt nachweisen, welche Art von Veränderungen im Alter als physiologisch anzusehen sind und welche nicht [2, 11]. Gerade beim älteren Patienten ist es wichtig, Immobilität und sekundäre Komplikationen infolge von Immobilität zu vermeiden [8, 13].

Es wird innerhalb der Leitlinien davon ausgegangen, dass es keine eindeutige Korrelation zwischen bildmorphologisch fassbaren Veränderungen und empfundenem Schmerz sowie empfundenen Symptomen gibt [1]. Die der Leitlinie zugrunde liegenden radiologischen Original- und Übersichtsarbeiten beziehen sich in weiten Teilen auf konventionelle Röntgenuntersuchungen der Wirbelsäule [1, 22].

Da wiederum bei einem pathoanatomisch bedingten Rückenschmerz eine zielgerichtete Therapie möglich ist, ist es wünschenswert, diese Patienten zu erkennen und entsprechend zu behandeln.

Um hier eine möglichst genaue und valide Aussage treffen zu können, ist die MRT-Untersuchung der röntgenmorphologischen Bildgebung nicht nur aus strahlenhygienischen Gründen klar vorzuziehen. Die MRT kann sowohl den Turgorverlust der Disci intervertebrales, eine Herniation der Disci intervertebrales, daraus resultierende Nervenaffektionen sowie auch osteochondrotische Veränderungen und Spondylarthrosen gut darstellen und quantifizieren [15, 18].

Ziel der vorgestellten Arbeit ist es, MR-morphologische Veränderungen zu zeigen, die regelhaft mit dem Älterwerden zu erwarten sind. Der zweite Fokus wird auf die degenerativen Veränderungen gelegt, die eine Korrelation zu der beklagten Beschwerdesymptomatik sowie zur Stärke der beklagten Schmerzsymptomatik des Patienten zeigen. Der dritte Fokus liegt auf den Auswirkungen des Rückenschmerzes und der daraus resultierenden Reaktion des Patienten: Nach welcher Zeit wird Kontakt zum Arzt aufgenommen, welche Beeinträchtigungen entstehen durch den Rückenschmerz im Alltag? Die erhobenen Parameter werden jeweils für die Gruppe der jungen Patienten (<65 Jahre) und der älteren Patienten (≥65 Jahre) analysiert und verglichen.

## Methode

Im Zeitraum vom 01.03.2018 bis zum 01.12.2020 wurde nach positivem Votum der Ethik-Kommission der Universität Witten/Herdecke eine prospektive Studie durchgeführt. Teil dieser Studie war die Untersuchung des Schmerzempfindens bei Rückenschmerzpatienten in 2 radiologischen Praxen der Maximalversorgung in einem deutschen Ballungsgebiet. In diese Studie wurden insgesamt 1047 Patienten eingeschlossen, die zu einer geplanten MRT-Untersuchung der Wirbelsäule kamen und bereit waren, an der Untersuchung teilzunehmen.

Die Patienten wurden gebeten, jeweils ihren aktuellen Schmerzscore auf einer visuellen Analogskala von 0 bis 10 anzugeben bzw. zu markieren. „0“ bedeutet dabei „kein Schmerz“, „10“ bedeutet „stärkster vorstellbarer Schmerz“. Ausgeschlossen wurden alle Patienten, bei denen ein Malignom in der Anamnese vorlag, alle Patienten, die schon an der Wirbelsäule operiert worden waren, sowie alle Patienten, die zum Zeitpunkt der Befragung oder bis zu 12 h davor eine Schmerzmedikation eingenommen hatten.

Es wurde jeweils ein Kurzinterview durchgeführt, in dem die Patienten nach der Beeinträchtigung im Alltag sowie nach der Beeinträchtigung in Freizeit und Familie durch den Rückenschmerz befragt wurden. Der Patient musste hier jeweils einen Wert zwischen 0 und 10 markieren. „0“ bedeutet „keine Beeinträchtigung“, „10“ bedeutet „stärkste vorstellbare Beeinträchtigung“. Es wurde nach dem Zeitraum zwischen Auftreten der Rückenschmerzen und dem ersten Arztkontakt bezüglich der Rückenschmerzen gefragt, hier gab der Patient jeweils den Zeitraum in Wochen an. Zusätzlich wurde gefragt, wie viele Tage seit Beginn der Rückenschmerzen es nicht möglich war, eine angemessene Aktivität (egal, ob beruflich oder im privaten Leben) auszuführen. Als weitere Parameter für die Auswertung wurden erhoben: Alter, Geschlecht, BMI, Grad der Facettengelenkarthrose, Grad der Osteochondrose nach Modic [18], Grad der spinalen Einengung in Prozent, Bestehen einer Nervenwurzelaffektion, Grad der Spondylolisthesis, falls vorhanden.

Alle statistischen Berechnungen wurden mittels multipler Regressionsanalyse in SPSS V27 (IBM, USA) durchgeführt; als statistisches Signifikanzniveau wurde *p* < 0,05 gesetzt.

## Ergebnisse

### Gesamtes Kollektiv

Eingeschlossen wurden 1047 Patienten: 448 Männer (42,8 %) und 599 Frauen (57,2 %). Das Durchschnittsalter der Gesamtgruppe betrug 55,9 Jahre (18 bis 92 Jahre). Das Alter der Männer betrug 56,2 Jahre (18 bis 92 Jahre), das der Frauen betrug 55,7 Jahre (18 bis 91 Jahre).

Der durchschnittliche BMI betrug 28,6 kg/m^2^ (Std.-Abw.: 5,9). Der durchschnittliche BMI der Männer betrug 28,8 kg/m^2^ (Std.-Abw.: 5,2), der der Frauen 28,5 kg/m^2^ (Std.-Abw.: 6,5).

Der durchschnittliche Schmerzscore der Gesamtgruppe betrug 5,34 (Std.-Abw.: 2,22). Der der männlichen Patienten war mit 5,09 (Std.-Abw.: 2,27) geringer ausgeprägt als der der weiblichen Patienten mit 5,54 (Std.-Abw.: 2,17).

Im gesamten Kollektiv konnte eine signifikante Korrelation zwischen Schmerzscore und Nervenwurzelaffektion gezeigt werden. Für die rezessale Nervenwurzelaffektion war die Korrelation mit +0,215 stärker ausgeprägt als für die neuroforaminale mit +0,142. Ebenfalls positiv korreliert waren die Facettengelenkarthrose (+0,166) und die spinale Enge (+0,201). Die übrigen untersuchten Variablen zeigten keine signifikante Korrelation.

Bei den Patientinnen zeigte sich eine signifikante positive Korrelation zwischen Schmerzscore und rezessaler Nervenwurzelaffektion mit 0,253, ebenfalls eine positive Korrelation von 0,218 zeigte sich zur spinalen Enge. Wie auch im Gesamtkollektiv zeigte auch die Facettengelenkarthrose mit 0,166 eine positive Korrelation zum Schmerzscore.

Bei den männlichen Patienten zeigte sich eine signifikante positive Korrelation zwischen Schmerzscore und rezessaler Nervenwurzelaffektion mit +0,189. Alle anderen Variablen erreichten kein Signifikanzniveau (Tab. 1).

**Table 1:** 

	Facettengelenkarthrose	Spinale Einengung	Osteochondrose	Rezessale Nervenwurzelaffektion	Intraforaminale Nervenwurzelaffektion
*Gesamt*	0,166	0,201	n. s.	0,215	0,142
*Männlich*	n. s.	n. s.	n. s.	0,189	n. s.
*Weiblich*	0,166	0,218	n. s.	0,253	n. s.

*n.* *s.* nicht signifikant

### Veränderungen im Alter

**Figure 1:**
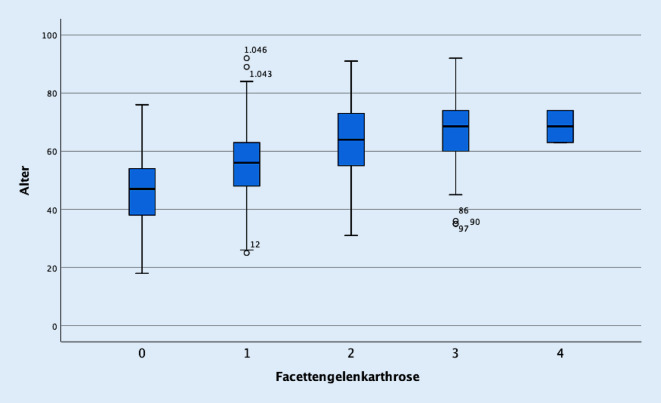

**Figure 2:**
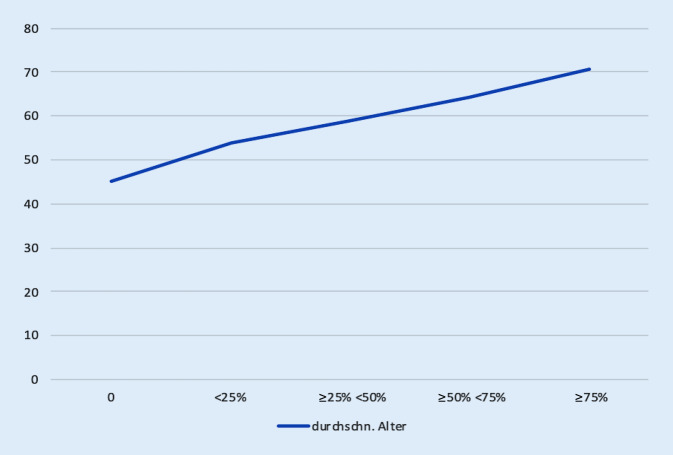

Es waren 284 Patienten 65 Jahre oder älter, entsprechend 763 jünger als 65 Jahre. In der Gesamtgruppe zeigte sich eine stark positive Korrelation zwischen Alter und Facettengelenkarthrose von +0,531 (Abb. 1). Ebenso zeigte sich eine positive Korrelation zwischen Alter und dem Grad der spinalen Einengung mit 0,267 und osteochondrotischen Veränderungen mit 0,378 (Abb. 2). Die intraforaminale Nervenwurzelaffektion war ebenfalls positiv mit dem Alter korreliert mit 0,257.

In der Gruppe der männlichen Patienten zeigte sich eine stark signifikante positive Korrelation zwischen Alter und dem Grad der Facettengelenkarthrose mit 0,516. Eine positive Korrelation konnte auch zwischen osteochondrotischen Veränderungen und Alter mit +0,372 festgestellt werden; für die anderen Variablen konnte kein signifikanter Zusammenhang nachgewiesen werden.

In der Gruppe der weiblichen Patienten zeigte sich eine signifikante positive Korrelation zwischen Alter und Grad der Facettengelenkarthrose mit +0,541, ebenso zwischen osteochondrotischen Veränderungen und Alter mit +0,383; für die übrigen untersuchten Variablen konnte keine signifikante Korrelation nachgewiesen werden (Tab. 2).

**Table 2:** 

	Facettengelenkarthrose	Spinale Einengung	Osteochondrose	Rezessale Nervenwurzelaffektion	Intraforaminale Nervenwurzelaffektion
*Gesamt*	0,531	0,267	0,378	n. s.	0,257
*Männlich*	0,516	n. s.	0,372	n. s.	n. s.
*Weiblich*	0,541	n. s.	0,383	n. s.	n. s.

*n.* *s.* nicht signifikant

Der ältere Patient mit Rückenschmerzen ging mit durchschnittlich 10 Wochen Wartezeit signifikant früher zum Arzt als der jüngere Patient mit durchschnittlich 17 Wochen. Zudem zeigte sich eine signifikant höher empfundene Beeinträchtigung im Alltag durch den Rückenschmerz beim älteren Patienten mit einem Score von 7,3 als beim jüngeren Patienten mit einem Score von 5,7. Bei allen übrigen Parametern zeigten sich zwischen den 2 Altersgruppen keine signifikanten Unterschiede.

### Vergleich der Gruppen mit dem geringsten (SC = 1) und dem höchsten angegebenen Schmerz (SC = 10)

Die Patienten in der Gruppe mit dem geringsten Schmerzniveau (*n* = 23) zeigten einen durchschnittlichen BMI von 27,69 (Std.-Abw.: 5,094) bei einem durchschnittlichen Lebensalter von 59,74 Jahren (35 bis 79 Jahre).

Die Patienten mit dem höchsten Schmerzniveau (*n* = 23) zeigten einen durchschnittlichen BMI von 31,00 (Std.-Abw.: 5,829) bei einem durchschnittlichen Lebensalter von 53,39 Jahren (31 bis 80 Jahre). Der durchgeführte T‑Test zeigte eine Signifikanz von 0,047 für den BMI, für das Alter keine Signifikanz mit 0,107.

### Lokalisationen

**Figure 3:**
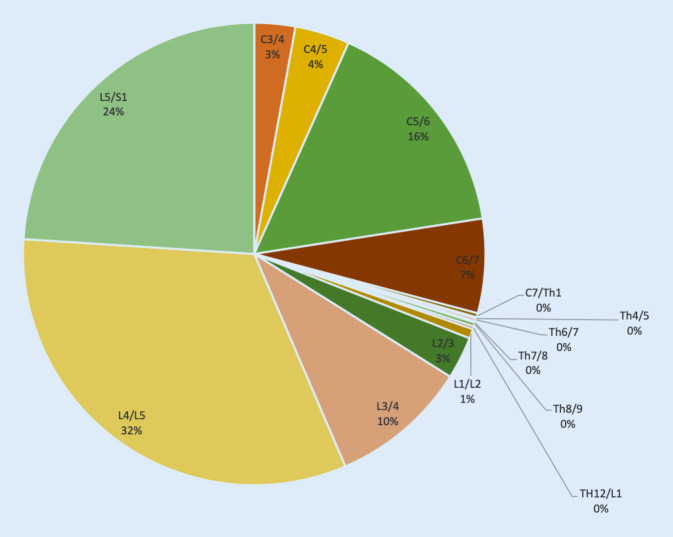

Als Hauptorte der Degeneration wurden das Segment L4/5 (32,5 %), das Segment L5/S1 (24,0 %) und das Segment C5/6 (15,6 %) diagnostiziert. Bei 72,1 % aller untersuchten Patienten fand sich die stärkste Ausprägung der Degeneration an diesen 3 Lokalisationen (Abb. 3). Bezüglich der Lokalisationen zeigte sich keine signifikante Abhängigkeit zum Lebensalter.

## Diskussion

Es zeigt sich keine Korrelation zwischen Alter und Schmerzscore. Ein Älterwerden bedeutet damit nicht, mehr Schmerzen an der Wirbelsäule zu haben, obwohl unsere Daten sehr wohl konkordant zur Literatur eine Veränderung der Wirbelsäule mit dem Alter aufzeigen [23]. Es zeigen sich im Verlauf der Zeit signifikante und stark positiv korrelierte Veränderungen an den Facettengelenken sowie osteochondrotische Veränderungen; dies ist vergleichbar zu den Ergebnissen anderer Untersuchungen [7, 19]. Die Spondylolisthesis ist in unserem Patientengut mit 0,3 % geringer ausgeprägt als in der Literatur, es zeigt sich aber wie in anderen Arbeiten eine positive Korrelation zu begleitenden strukturellen Schäden des Discus intervetebralis in den Segmenten mit der führenden Facettegelenksarthrose, also L4/5 und L5/S1 [7, 19]. Osteochondrotische Veränderungen, insbesondere in den Stadien 2 und 3, zeigen ebenfalls eine deutliche Zunahme mit dem Alter [3].

Korrelierend zu den oben genannten Untersuchungen zeigt unsere Studie eine Zunahme der Spondylarthrose mit dem Alter. Diese ist signifikant ausgeprägt und zeigt regelhafte degenerative Veränderungen an den Facettengelenken ab dem 56. Lebensjahr (Abb. 1). Im weiteren Verlauf zeigt sich eine kontinuierliche Zunahme der degenerativen Veränderungen der Facettengelenke mit dem Alter mit einem Plateau ab dem 68. Lebensjahr, wobei eine destruierende Facettengelenkarthrose in unserem Patientengut sehr selten vorkommt.

Auch eine progrediente Einengung des Spinalkanals infolge des Älterwerdens wird beschrieben [10]. Die in dieser Untersuchung beschriebene Verteilung basiert allerdings auf einer nur sehr kleinen Patientengruppe mit 98 Patienten und wird in der aktuellen Literatur durchaus kritisch hinterfragt [11]. Für die spinale Einengung zeigt die vorliegende Untersuchung eine signifikante Korrelation mit dem Alter des Patienten. In höherem Alter tritt die spinale Einengung signifikant öfter auf als in einem jüngeren Alter. Zudem zeigen die erhobenen Daten sowohl für die Spondylarthrose als auch für die spinale Einengung eine signifikante Korrelation zum angegebenen Schmerzscore.

Damit ist zu diskutieren, ob eine im Alter gehäuft vorkommende Veränderung gleichzeitig auch einen signifikanten Grund für Beschwerden darstellt. Die hypertrophe Spondylarthrose führt rein pathoanatomisch zu einer Einengung sowohl der Neuroforamina als auch des Spinalkanals [6].

Damit ist rein anatomisch zu erwarten, dass es infolge der spinalen Einengung und der fortschreitenden häufig hypertrophen Arthrose der Facettengelenke konsekutiv auch zu einer vermehrten Nervenwurzelaffektion kommt.

Dies bestätigen unsere Daten: Die intraforaminale Affektion der Nervenwurzeln zeigt eine positive und signifikante Korrelation mit dem Alter. Es zeigt sich darüber hinaus eine signifikante und deutlicher ausgeprägte Korrelation zwischen Schmerzscore und intraspinaler Nervenwurzelaffektion gegenüber der rezessalen (0,215 vs. 0,142). Die positive Korrelation zwischen Alter und intraforaminaler Nervenwurzelaffektion lässt sich wiederum durch die verstärkte und nahezu regelhaft mit dem Alter auftretende Spondylarthrose erklären. Auch die Literatur akzeptiert die Spinalkanalstenose als häufig auftretendes Merkmal des älteren Menschen, häufig korreliert mit einer begleitenden oder auch daraus resultierenden Schmerzsymptomatik [5, 14].

Eine CT-Untersuchung an 191 Patienten verglich das Auftreten der lumbalen spinalen Einengung mit dem Alter und zeigte – wie auch unsere Daten – ein gehäuftes Auftreten mit dem Alter. Gleichzeitig wurde ein dreifach erhöhtes Risiko nachgewiesen, an spezifischem Rückenschmerz zu leiden, wenn eine relevante lumbale spinale Einigung vorlag [12]. Diese Beobachtungen werden durch unsere Zahlen unterstützt: Auch in unserem Patientengut steigt die spinale Enge mit dem Alter an, und es zeigt sich gleichzeitig eine positive Korrelation zwischen Ausprägung der spinalen Enge und Alter.

Der ältere Patient empfindet die Beeinträchtigung durch den Rückenschmerz stärker als der jüngere Patient und sucht signifikant früher den Arzt auf. Es ist sinnvoll, dass der Patient mit spezifischem Rückenschmerz sich frühzeitig in eine zielgerichtete Behandlung begibt, um eine Chronifizierung zu vermeiden [9]. Eine zu frühe Bildgebung wird als nicht zielführend angesehen, da es häufig zu einer spontanen Remission der Rückenschmerzen kommen kann. In Deutschland wird gemäß S3-Leitlinie nach 4 bis 6 Wochen leitliniengerechter Behandlung empfohlen, die Indikation zur Bildgebung zu überprüfen und diese dann ggf. zu indizieren, wenn nicht Hinweise auf eine akute und bedrohliche Pathologie zu einer sofortigen Bildgebung mahnen [20]. In beiden Gruppen wird dieses empfohlene Zeitintervall deutlich überschritten, insofern zeigt sich hier keine verfrühte oder überdimensionierte Behandlungsbereitschaft in unserem Patientengut. Im Vergleich zu anderen Untersuchungen ist zu beachten, dass es sich bei unseren Patienten – gerade bei den älteren Patienten – jeweils um mobile und selbstständige Patienten handelt, die ambulant zu uns zur Diagnostik und ebenfalls ambulant zu ihrem behandelnden Arzt und in die weitere Therapie gegangen sind. Dies ist gerade beim geriatrischen Patienten nicht selbstverständlich und kann deshalb nicht auf die Gruppe der nicht mehr selbstständigen geriatrischen Patienten übertragen werden [23].

Wichtig erscheint nach den hier gefundenen Ergebnissen eine enge Korrelation zur Klinik des Patienten – egal, ob beim älteren oder jüngeren Patienten. In beiden Patientengruppen ist es wichtig, eine Chronifizierung zu vermeiden und dem Patienten über eine zielgerichtete und möglichst an die persönliche Situation angepasste Therapie eine Rückkehr in sein normales Leben zu ermöglichen.

### Limitationen

Die von uns vorgestellte Untersuchung legt bewusst einen Fokus auf die degenerativ bedingten Veränderungen der Wirbelsäule beim ambulanten, mündigen Patienten. Gerade bei den geriatrischen Patienten gibt es zahlreiche, die aufgrund von Vorerkrankungen oder geistigen Einschränkungen nicht mehr ambulant behandelt werden können. Hier bedarf es einer gesonderten Untersuchung dieser Gruppe. Die Anzahl an Patienten mit unspezifischem Rückenschmerz ist hoch, bei den älteren Patienten aber in der Tendenz abnehmend, dennoch sollte auch für diese Gruppe eine entsprechende Untersuchung erfolgen.

## Zusammenfassung

Die von uns durchgeführte Studie zeigt eine hochsignifikante und stark positive Korrelation zwischen Facettengelenkarthrose und Alter der Patienten (+0,531). Eine mittlere Korrelation zeigt sich zwischen osteochondrotischen Veränderungen und dem Alter (+0,387). Eine geringe, aber messbare Effektstärke mit hoher Signifikanz zeigt sich zwischen Alter der Patienten und spinaler Enge (+0,267) sowie intraforaminaler Nervenwurzelaffektion (+0,257). Der ältere Patient leidet nicht unter größeren Schmerzen als der jüngere Patient. Der an Rückenschmerz leidende ältere Patient zeigt eine signifikant höhere Beeinträchtigung seiner Alltagsaktivitäten und sucht früher, aber im Mittel noch nicht früh genug, den behandelnden Arzt auf.

## Fazit für die Praxis

- Es besteht keine Korrelation zwischen Lebensalter und Schmerz.
- Rückenschmerz führt beim älteren Patienten zu einer signifikanten Beeinträchtigung im Alltag.
- Der ältere Patient sucht signifikant früher, nach durchschnittlich 10 Wochen, ärztlichen Rat. Bezogen auf die aktuellen Leitlinien ist das aber deutlich zu spät.
- Die am häufigsten mit dem Alter auftretende und regelhaft im Alter zunehmende Veränderung ist die Facettengelenkarthrose.
- Positiv mit dem Alter korreliert und regelhaft mit dem Alter zunehmend finden sich osteochondrotische Veränderungen, spinale Einengungen und intraforaminale Nervenwurzelaffektionen.
- Signifikante Korrelationen zeigen sich zwischen empfundenem Schmerz und rezessaler sowie intraforaminaler Nervenwurzelaffektion, Facettengelenkarthrose und spinaler Enge.
